# Comparison of Biological, Pharmacological Characteristics, Indications, Contraindications and Adverse Effects of JYNNEOS and ACAM2000 Monkeypox Vaccines

**DOI:** 10.3390/vaccines10111971

**Published:** 2022-11-21

**Authors:** Sultan Ayoub Meo, Abeer A. Al-Masri, David C. Klonoff, Abdullah Nasser Alshahrani, Thamir Al-khlaiwi

**Affiliations:** 1 Department of Physiology, College of Medicine, King Saud University, Riyadh 11461, Saudi Arabia; 2 Diabetes Research Institute, Mills-Peninsula Medical Center, San Mateo, CA 94401, USA

**Keywords:** monkeypox, vaccines, JYNNEOS, ACAM2000, pharmacology, adverse effects

## Abstract

Human monkeypox is an emerging viral zoonotic disease, that has caused highly distinctive, challenging and threatening problems worldwide. The US Food and Drug Administration (FDA) has given interim authorization for the JYNNEOS and ACAM2000 vaccines for the outbreak of monkeypox 2022. The present study aims to highlight the globally derived evidence about the biological and pharmacological features, indications, contraindications and adverse effects of JYNNEOS and ACAM2000 vaccines. Initially, 82 documents were selected and, finally, 14 fact sheets, documents and international organizations were included. The data were recorded from the World Health Organization (WHO), Centers for Disease Control and Prevention (CDC), Food and Drug Administration (FDA) USA, ISI-Web of Science, PubMed, EMBASE and Scopus. The data revealed that the JYNNEOS vaccine has been recommended to children, adults, females during pregnancy and people of all age groups with a dose of 0.5 mL, and the complete vaccination cost per person is about USD 115. It provides immunogenicity, and the mean titer of neutralizing antibodies was 153.5. However, the ACAM2000 vaccine is contraindicated in infants and pregnant females, and recommended to people over 18 years of age and older, with a single dose of 0.0025 mL, and a cost of about USD 139. ACAM2000 provides immunogenicity, and the mean titer of neutralizing antibodies was 79.3. The JYNNEOS vaccine has mild adverse effects including pain, redness, swelling or itching at the site of the vaccine shot, fever, fatigue, headache, nausea and muscle pain. However, the ACAM2000 vaccine can cause pain, redness, edema, headache, fever, fatigue, muscle pain, body ache, nausea, vomiting, diarrhea, shortness of breath and increased risk of myopericarditis and cardiomyopathy. The evidence supports the view that both vaccines are beneficial, but the overall impact of JYNNEOS is better than that of ACAM2000.

## 1. Introduction

Human monkeypox is an emerging viral zoonotic disease that is causing a threat to the global healthcare system. The spread of the disease occurs due to its various epidemiology and transmission trends. Viral infections are highly contagious and provoke numerous health and socio-economic harms [1]. This year, from 1 January 2022 to 4 November 2022, the virus swiftly spread from endemic to non-endemic regions, involving 109 countries, and infecting 78,229 people; 928 people were from seven endemic countries, and 77,301 cases were in 102 non-endemic countries in Europe, America, Australia and Asia [2]. The spread of monkeypox infection is linked to various challenging factors including physical and sexual contact, skin lesions, body fluids, respiratory droplets and a contagious environment [2].

The chief strategy for prevention is vaccination. However, no specific vaccines have been developed for MPV. The literature supports the idea that smallpox vaccines could be effective to protect people against the MPV [3].

The death rate from the current epidemic is lower than what might be expected from historical data. As of the present month, out of more than 78,000 people confirmed to have had monkeypox infections, at least 41 died, which made for a death rate of about 0.05% [4]. This rate is less than the historic death rate of 0–11% during prior monkeypox outbreaks, as reported by the World Health Organization [5]. Nevertheless, there is fear and concern about this epidemic and a safe and effective monkeypox vaccine is needed by people all over the world.

During the current situation of the spread of human monkeypox disease, people worldwide are facing major healthcare challenges because there is no specific treatment. Vaccination is the best tool to fight against such contagious situations [6]. On 9 August 2022, the US Food and Drug Administration (FDA) [7] provided interim authorization for the use of JYNNEOS [7] and ACAM2000 vaccines [8] to prevent human monkeypox disease. The monkeypox-specific vaccination recommendation was achieved in a short period and many people have expressed some concerns about these two FDA interim-authorized vaccines [7,8].

The overall risk–benefit balance of monkeypox-specific vaccination still needs further evidence-based clarification [6]. This study investigates the global evidence comparing biological and pharmacological characteristics, indications, contraindications and adverse effects of JYNNEOS and ACAM2000 vaccines.

## 2. Materials and Methods

This study was performed in the Department of Physiology, College of Medicine, King Saud University, Riyadh, Saudi Arabia. In the present study, we obtained data from worldwide highly reliable and evidence-based organizations, and websites which highlight the biological and pharmacological findings, indications, contraindications and side effects of JYNNEOS and ACAM2000 vaccines. The data were recorded from the World Health Organization (WHO) [9], US Food and Drug Administration (FDA) [10], Centers for Disease Control Prevention (CDC) [11], Clarivate Analytics Web of Science [12], Medline, EMBASE, PubMed, [13] and Scopus. The data were searched by using key terms including monkeypox, vaccines, JYNNEOS, ACAM2000, pharmacological characteristics, indications, contraindications, adverse events, etc.

**Figure 1:**
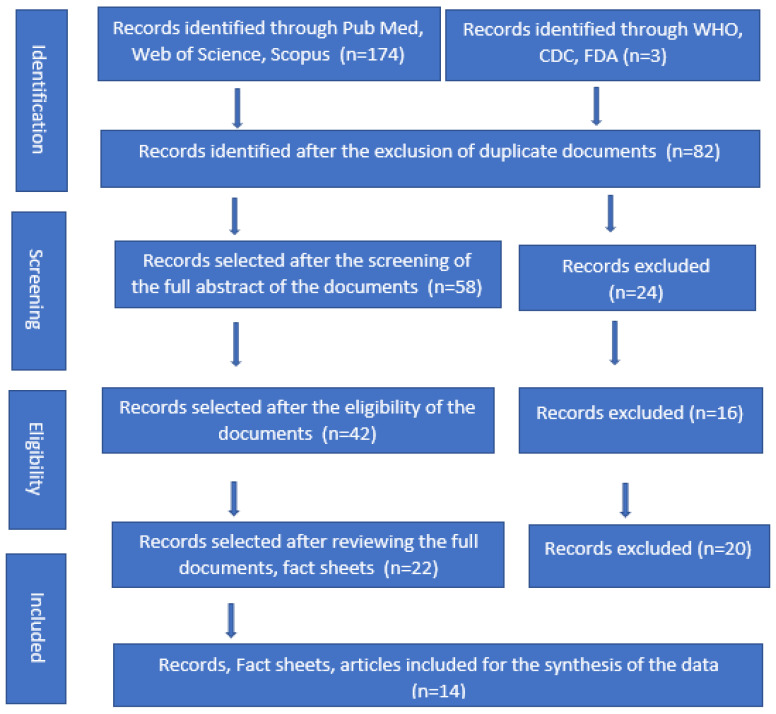
PRISMA flow diagram for the selection of organizations, fact sheets and documents about JYNNEOS and ACAM2000 monkeypox vaccines.

Initially, 174 documents and 3 international health organizations were identified through the systematic databases, PubMed, Web of Science, Scopus and Google Scholar. A total of 82 documents were reviewed, and finally, we included data from 14 articles, facts sheets and international organizations’ websites, including the World Health Organization (WHO), US Food and Drug Administration (FDA), Centers for Disease Control Prevention (CDC) (Figure 1). The international organizations, literature and weblinks in which JYNNEOS and ACAM2000 vaccines were mentioned were eligible for inclusion. There were no specific limitations on study design, type or publication language. The required information was obtained from the selected organizations and documents. Two co-authors reviewed the literature, recorded the information and entered the findings in tabular form. After that, another research member rechecked the findings.

Ethics statement: The data were recorded from international organizations and publicly available databases on monkeypox and JYNNEOS and ACAM2000 vaccines, hence, ethical approval was not required.

## 3. Results

Table 1 demonstrates the comparison between biological and pharmacological characteristics of JYNNEOS and ACAM2000 vaccines. The US Food and Drug Administration (FDA) has granted emergency use authorization for the administration of the JYNNEOS and ACAM2000 vaccines. The JYNNEOS vaccine has been recommended for children, adults, females during pregnancy and people of all age groups with a dose of 0.5 mL. A complete two-part series of injected vaccinations costs about USD 115 per person. It provides immunogenicity after the second dose of vaccination (Table 1). The mean titer of neutralizing antibodies was 153.5 (Table 2).

The JYNNEOS vaccine has mild side effects including “pain, redness, swelling or itching at the site of the vaccine injection, fever, fatigue, headache, muscle pain and nausea” (Table 2).

The ACAM2000 vaccine is contraindicated in infants and pregnant females, and recommended to people over 18 years of age and older, with a single dose of 0.0025 mL, and a cost of about USD 139 [18]. ACAM2000 provides immunogenicity, and the mean titer of neutralizing antibodies was 79.3 [19] (Table 2). The ACAM2000 vaccine can cause pain, redness, swelling and itching at the site of injection, fatigue, fever, headache, muscle pain, body aches, nausea, vomiting, diarrhea, lymph node enlargement and shortness of breath. Moreover, there are some severe adverse effects including an increased risk of myopericarditis and cardiomyopathy reported for the ACAM2000 vaccine (Table 2). The available evidence supports the conclusion that both vaccines have adverse effects, but the overall impact of JYNNEOS, compared to ACAM2000, is more favorable.

**Table 2:** Comparison of immunogenicity, contraindication and adverse effects between JYNNEOS and ACAM2000 vaccines.

Characteristics	JYNNEOS Vaccine	ACAM2000 Vaccine
Immunogenicity/duration of immunity	Two weeks after the second dose of JYNNEOS [20]	Four weeks after the single dose of ACAM2000 [17]
Neutralizing antibodies	At day 14, the mean titer of neutralizing antibodies was 16.2 [19]. At week 6, the mean titer of neutralizing antibodies was 153.5 [19]	At day 14, the mean titer of neutralizing antibodies by ACAM2000 was 16.2 [19]. At week 4, the mean titer of neutralizing antibodies was 79.3 [19]
Risks in pregnancy, infancy and in children	JYNNEOS may be administered during pregnancy, breastfeeding and infancy [8]. This vaccine is off-label for children and pregnant women [15,20]	ACAM2000 has a risk of adverse effects and is contraindicated during pregnancy, breastfeeding and in infants [8,17]
Contraindication	For the emergency uses of JYNNEOS, no contraindications are identified. It is contraindicated for people with a history of atopic dermatitis, an allergy to one of the vaccine components. JYNNEOS is safe in persons with immunocompromising conditions [8,16,20]	Pregnancy, breastfeeding, smoking, hypertension, diabetes mellitus, coronary artery disease, cardiomyopathy, people with allergic reactions and anaphylaxis. ACAM2000 is contraindicated in people with immunosuppression conditions, e.g., “leukaemia, lymphoma, human immunodeficiency virus infection, and acquired immune deficiency syndrome (AIDS)” [16,17].
Common adverse effects	Local: pain, redness, swelling, itching, hyperpigmentation, skin discoloration Systemic: fatigue, headache, myalgias, nausea, chills, fever [15,20]	*Local:* pain, redness, swelling and itching at the site of injection. *Systemic:* fatigue, fever, muscle pain, headache, lymph node enlargement, malaise, arm soreness, body aches, skin rash, nausea, vomiting, diarrhea, swelling of the face, dizziness and shortness of breath [16,17]
Severe adverse effects	No risk of severe adverse effects such as myopericarditis or cardiomyopathy in recipients of JYNNEOS [15,20]	Increased risk of myopericarditis and cardiomyopathy [16,17]

## 4. Discussion

Human monkeypox is an emerging viral zoonotic disease, potentially threatening the state of global health [1]. This year, from 1 January 2022 to 4 November 2022, the virus has shown various transmission trends and rapidly spread from historically endemic to non-endemic regions, involving 109 countries, and infecting 78,229 people; 928 people were from seven endemic countries, and 77,301 cases were in 102 non-endemic countries in Europe, America, Australia and Asia [2]. The primary strategy for prevention and transmission of the monkeypox infection would be vaccination. In August 2020, the US Food and Drug Administration (FDA) provided emergency authorization for the JYNNEOS and ACAM2000 vaccines [7,8]. There is reasonable evidence that both JYNNEOS and ACAM2000 vaccines may be effective to fight against the monkeypox disease.

The effectiveness of both JYNNEOS and ACAM2000 in various age groups was analyzed. JYNNEOS is well acceptable for active immunization for the prevention of monkeypox disease in individuals less than 18 years, and 18 years of age and older [14]. However, it has been reported that the ACAM2000 vaccine is contraindicated in infants, mainly those aged <1 year (Table 1) [16]

Pittman et al. (2019) [19] conducted a clinical trial and assessed the efficacy of neutralizing antibodies of vaccinia Ankara (MVA) and ACAM2000. MVA vaccination induced a mean titer of neutralizing antibodies of 153.5 at week 6, as compared with 79.3 at week 4 with ACAM2000. At day 14, the mean titer of neutralizing antibodies with a single MVA vaccination was 16.2, equal to the 16.2 for ACAM2000 [19].

In terms of the risk factors of these two vaccines during pregnancy and infancy and in children, JYNNEOS demonstrated good acceptability, and this vaccine can be administered during pregnancy, breastfeeding and infancy [8]. However, ACAM2000 is associated with a risk of developing adverse effects during pregnancy and breastfeeding and in infants (Table 2) [16,17]. 

The JYNNEOS vaccine costs less than the ACAM2000 vaccine. Their costs are USD 115.5 and USD 139 per person, respectively [18]. While a few developed nations are providing vaccination to their citizens free of cost, many are passing along the cost. The public must advocate that the vaccine should be affordable for all citizens because the health and lives of all individuals in a society are important. Special consideration must be taken by low-income countries to provide vaccines to their citizens. JYNNEOS vaccines would likely be preferred because of their low cost, but the type of storage requirement is also a consideration. Despite its lower cost, JYNNEOS vaccine is stored at a temperature from −15 °C to −25 °C, which is the same temperature at which the ACAM2000 vaccines must be stored [15].

Again, this poses a distribution challenge in low-income countries, because in many countries there is an energy crisis to manage standard storage conditions, including freezers, to ensure the promising results of both vaccines. The JYNNEOS vaccine has a low cost and better benefits.

Rao et al. (2022) [16] reported a low level of certainty about some adverse events occurring following JYNNEOS compared with ACAM2000. The ACAM2000 vaccine causes serious adverse effects including myopericarditis, however, these effects are not reported when the public receives JYNNEOS vaccines. We believe that the literature favors the JYNNEOS vaccine as a preferable alternative over ACAM2000 for vaccination.

The pharmacological characteristics and contraindications of both vaccines were studied. The available data on the emergency uses of JYNNEOS did not identify contraindications. However, on the safe side, it is contraindicated for people with a history of atopic dermatitis, an allergy to one of the vaccine components. JYNNEOS is safe in people with immunocompromising conditions [8]. However, the ACAM2000 vaccine is contraindicated during pregnancy and breastfeeding and for people with a known history of hypertension, diabetes mellitus, coronary artery disease and cardiomyopathy. The ACAM2000 vaccine is also contraindicated for people with moderate to severe allergic reactions, who are suffering from chronic debilitating diseases such as lymphoma, leukemia, human immunodeficiency virus infection and acquired immune deficiency syndrome [16,17].

In the present study, the severe adverse effects of JYNNEOS and ACAM2000 vaccines were investigated. There was no risk for severe adverse effects such as myopericarditis or cardiomyopathy reported among the recipients of JYNNEOS [20,21]. However, ACAM2000 vaccines can cause severe adverse effects including difficulty in breathing, swelling of the face, dizziness and an increased risk of myopericarditis and cardiomyopathy [16,17].

More recently, Kuehn (2022) [22] reported that the virus used in the development of the JYNNEOS vaccine cannot replicate, and the has limited contraindications compared to ACAM2000. However, the ACAM2000 vaccine contains a live vaccinia virus that can replicate and cause infections in vaccinated individuals or be transmitted to others [21].

Payne et al. (2022) [23] conducted a study on JYNNEOS vaccines and examined the occurrence of monkeypox disease in unvaccinated people and people who had received a single dose of the JYNNEOS vaccine among males aged 18–49 years in mid-2022. The monkeypox occurrence was 14 times higher among the unvaccinated males compared to those people who had received the first dose of the JYNNEOS vaccine. These findings indicate that a single dose of the JYNNEOS vaccine can protect people against monkeypox infection.

ACAM2000 and JYNNEOS are two US FDA-approved vaccines, but ACAM2000 may cause some serious adverse effects including cardiac problems, whereas JYNNEOS has limited adverse effects. Therefore, JYNNEOS is more acceptable than ACAM2000 [8].

According to the CDC, the JYNNEOS vaccine is approved for the prevention of monkeypox and smallpox disease, whereas the ACAM2000 vaccine is approved for immunization against smallpox disease and made available for use against monkeypox under an FDA Expanded Access Investigational New Drug (EA-IND) protocol. However, JYNNEOS is recommended for pre-exposure prophylaxis against orthopoxvirus infection among people at risk of exposure to monkeypox disease [16]. The present outbreaks have highlighted the acute need for continuous monitoring of the outcomes of the vaccination and therapeutic modalities to develop a safe, effective and new-generation vaccine specific to monkeypox disease.

## 5. Study Strengths and Limitations

This study added to the literature to highlight the global pieces of evidence about the comparison of biological and pharmacological features, indications, contraindications and adverse effects of JYNNEOS and ACAM2000 vaccines for a better understanding of these two vaccines in the prevention of monkeypox disease. Moreover, the analysis of the findings is based on highly reliable international health organizations, and evidence-based documents. The limitation of this study is that, still, the literature is lacking information on these two vaccines’ impact on multinational populations worldwide.

## 6. Conclusions

The FDA has approved the use of JYNNEOS for the prevention of smallpox and monkeypox disease in infants, children and adults 18 years of age and older determined to be at high risk for smallpox or monkeypox infection. On the other hand, the ACAM2000 vaccine is approved for immunization against smallpox disease and has been made available for use against monkeypox under an Expanded Access Investigational New Drug (EA-IND) protocol. These vaccines can protect people from monkeypox disease through antibody formation, and attendant immunity against monkeypox infection. It is hoped that these vaccines will end the current outbreak of monkeypox disease. The evidence supports the conclusion that both vaccines are beneficial, but the overall impact of JYNNEOS is better than ACAM2000.

## Figures and Tables

**Table 1:** Comparison between biological and pharmacological characteristics of JYNNEOS and ACAM2000 vaccines.

Characteristics	JYNNEOS Vaccine	ACAM2000 Vaccine
Generic name	Smallpox and monkeypox vaccine live non-replicating	Smallpox vaccinia, vaccine, live replicating
Brand name	JYNNEOS/Imamune/Imvanex, MVA Vaccinia Ankara	ACAM2000 Emergent Bio Solutions
Types of vaccine	Third-generation vaccine [14]	Second-generation vaccine [14]
Manufacturer, city and country	Bavarian Nordic, Hørsholm, Denmark	Gaithersburg, MD, USA and Sanofi Pasteur Biologics, LLC [9]
Vaccine production	Prepared using weakened live vaccinia virus, cannot cause smallpox or monkeypox. Produced from “strain-modified vaccinia Ankara-Bavarian Nordic (MVA-BN), an attenuated, non-replicating orthopoxvirus” [2]	The vaccine is made from a virus called vaccinia is a “pox”-type virus [7]
Replication-competent	A live-attenuated, non-replicating vaccine [8]	A live-attenuated replicating vaccine [8]
Produced from	“Strain-modified Vaccinia Ankara-Bavarian Nordic (MVA-BN), an attenuated, non-replicating orthopoxvirus” [7]	ACAM2000 is a “live vaccinia virus derived by plaque purification from a previously calf lymph-produced vaccine (Dryvax) and manufactured in Vero cells” [9]
Effective in age groups	Active immunization for the prevention of monkeypox disease in individuals less than 18 years, and 18 years of age and older [15]	Not effective in infants mainly aged <1 year, although vaccination is contraindicated [16]
FDA approval	FDA granted an emergency use authorization (EUA) for the emergency use of JYNNEOS/interim authorized, on 9 August 2022 [7], and a supplementary letter was issued on 2 September 2022	Interim authorized 28 June 2022, for people aged 1 year and older for monkeypox prophylaxis, CDC, ACAM2000 vaccine [17]
Dose(s)	Age <18 years: subcutaneous injection, 2 doses (0.5 mL each) 4 weeks (28 days) apart. Age ≥18 years: intradermal injection, two doses (0.1 mL each) 4 weeks (28 days) apart [15]	A single dose of 0.0025 mL droplet of reconstituted vaccine [17]
Booster shots	“Booster dose is recommended every two years for people with high-virulent orthopoxvirus, and every ten years for those in contact with low-virulent strains” [8,16]	High risk of exposure, researchers working in laboratories handling “variola virus, and monkeypox virus” should receive a booster dose every three years [16]
Route of administration	Subcutaneous injection [15]	Need a trained person, percutaneous route (scarification), prick the skin several times with a droplet of the vaccine. ACAM2000 cannot be given through subcutaneous, intradermal, intramuscular or intravenous routes [17]
Vaccination cost	The complete vaccination cost per person is about USD 115 [18]	The complete vaccination cost per person is about USD 139 [18].
Storage	Stored at −15 °C to −25 °C, once thawed the vaccine may be kept at +2 °C to +8 °C for 8 weeks [15]	Stored at −15 °C to −25 °C, shipment at −10 °C. After reconstitution use within 6–8 h at room temperature 20–25 °C. Reconstituted vaccine stored at 2–8 °C no longer than 30 days [17]
Mechanism of action	Produces “humoral and cellular immune responses to orthopoxviruses, neutralizing antibody for the prevention of smallpox and monkeypox” [15]	ACAM2000 vaccine stimulates the immune system to develop antibodies and cells in the blood to fight against infection [10]

## Data Availability

May be provided on reasonable request to corresponding author.
